# Majorana charges, winding numbers and Chern numbers in quantum Ising models

**DOI:** 10.1038/s41598-017-08323-0

**Published:** 2017-08-15

**Authors:** G. Zhang, C. Li, Z. Song

**Affiliations:** 1 College of Physics and Materials Science, Tianjin Normal University, Tianjin, 300387 China; 2 School of Physics, Nankai University, Tianjin, 300071 China

## Abstract

Mapping a many-body state on a loop in parameter space is a simple way to characterize a quantum state. The connections of such a geometrical representation to the concepts of Chern number and Majorana zero mode are investigated based on a generalized quantum spin system with short and long-range interactions. We show that the topological invariants, the Chern numbers of corresponding Bloch band, is equivalent to the winding number in the auxiliary plane, which can be utilized to characterize the phase diagram. We introduce the concept of Majorana charge, the magnitude of which is defined by the distribution of Majorana fermion probability in zero-mode states, and the sign is defined by the type of Majorana fermion. By direct calculations of the Majorana modes we analytically and numerically verify that the Majorana charge is equal to Chern numbers and winding numbers.

## Introduction

Characterizing the quantum phase transitions (QPTs) is of central significance to both condensed matter physics and quantum information science. Exactly solvable quantum many-body models are benefit to demonstrate the concept and characteristic of QPTs. Recently, topological phases and phase transitions^1^ have attracted much attention in various physical contexts. In general, QPTs are classified two types, characterized by topologically nontrivial properties in the Hilbert space and by the local order parameters associated with symmetry breaking^2^, respectively. Both conventional and topological QPTs refer to the sudden change of the groundstate properties driven by the change of external parameters. A topological QPT involves the change of ground-state topological properties which are indicated by topological quantum discrete numbers^3, 4^, while the various phases in a conventional QPT are distinguished by continuously varying order parameters. The topological quantum number is topological invariant, such as Chern number and Majorana zero mode, which have been received much recent interest^5–17^.

In general, a conventional QPT is always associated with a spontaneously symmetry breaking, while a topological QPT is accompanied by a change of a topological numbers. Two different measures, order parameter and topological invariant quantity, are employed to differentiate the phase boundaries of two kinds of QPTs. Nevertheless, so far there are no evidences to suggest that the two types of QPTs are absolutely incompatible, which means they cannot occur at the same point for certain systems. An interesting question is whether the local order parameter and topological order parameter can coexist to characterize the quantum phase transitions. A promising connection between two types of QPTs is the Jordan-Wigner transformation, which maps a Ising chain to *two types of Kitaev chains* with even and odd parity of particle numbers, respectively. One of Kitaev chain and Ising chain share a common ground state, rather than the complete ground states in some regions, in which the degeneracy of ground states leads to spontaneous symmetry breaking for spin model. This duplex Kitaev model is responsible for the symmetry breaking when QPT occurs. In recent work^18^, it is shown that the variation of the groundstate energy density for a class of exactly solvable quantum Ising models, which is a function of a loop in a two-dimensional auxiliary space, experiences a nonanalytical point when the winding number of the corresponding loop changes. This fact indicates that this class of models can be joint ones in which a topological and a conventional QPTs occur simultaneously.

In this paper, we investigate topological properties in a family of exactly solvable Ising models with short- and long-range interactions. We introduce the concept of Majorana charge to indicate the phase diagram based on the corresponding Majorana tight-binding lattice with open boundary condition. The magnitude of Majorana charge is determined by the distribution of Majorana fermion probability in zero-mode states, while its sign is determined by the types of Majorana fermions. We show that the topological invariants, the Chern number of a corresponding Bloch band is equal to the winding number in the auxiliary plane. Furthermore, by direct calculations of the Majorana modes we analytically and numerically verify that the Majorana charge is equal to Chern numbers and winding numbers. These indicate that three quantities can equally characterize the phase diagram in quantum spin systems.

## Results

This work focuses on a class of quantum spin chains. To investigate the phase diagram and the feature of the quantum phases, we will subsequently take the Jordan-Wigner transformation, introduce pseudo-spin representation, and Majorana fermion representation. There are four kinds of systems involved, spin chain, spinless fermion, pseudo-spin, and Majorana fermion. We will take three steps: (i) spin chain→spinless fermion; (ii) spinless fermion→pseudo-spin (Winding and Chern numbers); (iii) spinless fermion→Majorana fermion (Edge modes and Majorana charge).

### Model and pseudo-spin representation

We consider a generalized one-dimensional quantum spin model, which was exactly solved four decades ago^19^. It contains long-range interactions and the Hamiltonian has the form

1 $$\begin{array}{rcl}H & = & \sum _{n=1}^{M}\sum _{j=1}^{N}\,({J}_{n}^{x}{\sigma }_{j}^{x}{\sigma }_{j+n}^{x}+{J}_{n}^{y}{\sigma }_{j}^{y}{\sigma }_{j+n}^{y})\\  &  & \times \prod _{l=j+1}^{j+n-1}\,{\sigma }_{l}^{z}+g\sum _{j=1}^{N}\,{\sigma }_{j}^{z}\end{array}$$

The operators ${\sigma }_{i}^{x,y,z}$ are the Pauli matrices for spin at *i*th site. In the case *M* = 1, it is reduced to an ordinary anisotropic *XY* model which has been employed as a platform to test the signatures of QPT, such as entanglement^20^, geometric phase^21, 22^, decoherence^23^, and fidelity^24^. In large *N* limit, $M\ll N$, the Hamiltonian can be diagonalized as the form

2 $$H=\sum _{k}\,{\varepsilon }_{k}\,({\gamma }_{k}^{\dagger }{\gamma }_{k}-\frac{1}{2}),$$

via a conventional Jordan-Wigner transformation

3 $${\sigma }_{j}^{z}=1-2{c}_{j}^{\dagger }{c}_{j},{\sigma }_{j}^{y}={\rm{i}}{\sigma }_{j}^{x}{\sigma }_{j}^{z},$$

4 $${\sigma }_{j}^{x}=-\prod _{l < j}\,(1-2{c}_{l}^{\dagger }{c}_{l})\,({c}_{j}+{c}_{j}^{\dagger }),$$

and Fourier transformation

5 $${c}_{j}=\frac{1}{\sqrt{N}}\sum _{k}\,{c}_{k}{e}^{ikj},$$

and a Bogoliubov transformation

6 $${c}_{k}={u}_{k}{\gamma }_{k}+i{v}_{k}{\gamma }_{-k}^{\dagger }.$$

Here *γ*_*k*_ is a fermion operator and the parameters are

7 $${u}_{k}=\,\cos \,\frac{{\theta }_{k}}{2},{v}_{k}=\,\sin \,\frac{{\theta }_{k}}{2},$$

with

8 $$\cos \,{\theta }_{k}=\frac{2}{{\varepsilon }_{k}}[g-\sum _{n=1}^{M}\,({J}_{n}^{x}+{J}_{n}^{y})\,\cos \,(nk)],$$

9 $$\sin \,{\theta }_{k}=\frac{2}{{\varepsilon }_{k}}\sum _{n=1}^{M}\,({J}_{n}^{x}-{J}_{n}^{y})\,\sin \,(nk).$$

The spectrum is in the form

10 $$\begin{array}{rcl}{\varepsilon }_{k} & = & 2\{{[\sum _{n=1}^{M}({J}_{n}^{x}-{J}_{n}^{y})\sin (nk)]}^{2}\\  &  & {+{[\sum _{n=1}^{M}({J}_{n}^{x}+{J}_{n}^{y})\cos (nk)-g]}^{2}\}}^{\mathrm{1/2}},\end{array}$$

where *k* ∈ [−*π*, *π*). Based on this analysis, the groundstate phase diagram can be obtained. Actually, for *k* = *k*_*c*_ = 0, we have

11 $${\varepsilon }_{{k}_{c}}=2\,|\sum _{n=1}^{M}\,({J}_{n}^{x}+{J}_{n}^{y})-g|.$$

And for *k* = *k*_*c*_ = *π*, we have

12 $${\varepsilon }_{{k}_{c}}=2\,|\sum _{n=1}^{M}\,{(-1)}^{n}\,({J}_{n}^{x}+{J}_{n}^{y})-g|.$$

We find that the derivatives of ${\varepsilon }_{{k}_{c}}$ with respect to parameters $\{{J}_{n}^{x},{J}_{n}^{y},g\}$ experience a discontinuity at points

13 $$g={g}_{c}=\sum _{n=1}^{M}\,({J}_{n}^{x}+{J}_{n}^{y}).$$

or

14 $$g={g}_{c}=\sum _{n=1}^{M}\,{(-1)}^{n}\,({J}_{n}^{x}+{J}_{n}^{y}).$$

In this paper, we will consider the phase diagram in alternative ways: pseudo spin and Majorana fermion representations. This starting point is the spinless fermion Hamiltonian

15 $$H={H}_{{\rm{ch}}}+{H}_{{\rm{b}}},$$

with

16 $$\begin{array}{rcl}{H}_{{\rm{ch}}} & = & \sum _{n=1}^{M}\,\sum _{j=1}^{N-n}\,[({J}_{n}^{x}+{J}_{n}^{y})\,{c}_{j}^{\dagger }{c}_{j+n}+({J}_{n}^{x}-{J}_{n}^{y})\,{c}_{j}^{\dagger }{c}_{j+n}^{\dagger }+{\rm{H}}.{\rm{c}}.]\\  &  & +\sum _{j=1}^{N}\,(g-2g{c}_{j}^{\dagger }{c}_{j}),\end{array}$$

and

17 $$\begin{array}{r}{H}_{{\rm{b}}}={(-1)}^{{N}_{p}+1}\sum _{n=1}^{M}\,\sum _{j=N-n+1}^{N}\,[({J}_{n}^{x}+{J}_{n}^{y})\,{c}_{j}^{\dagger }{c}_{j+n}+({J}_{n}^{x}-{J}_{n}^{y})\,{c}_{j}^{\dagger }{c}_{j+n}^{\dagger }]\,+\,{\rm{H}}.{\rm{c}}.,\end{array}$$

which represent the chain and the boundary parts, respectively. Here, ${N}_{p}={\sum }_{j=1}^{N}{c}_{j}^{\dagger }{c}_{j}$ is the number of fermion.

In large *N* limit and $M\ll N$, the Hamiltonian (15) can be written in *k* space as

18 $$\begin{array}{rcl}H & = & \sum _{k}\,\{2[\sum _{n=1}^{M}\,({J}_{n}^{x}+{J}_{n}^{y})\,\cos \,(nk)-g]\,{c}_{k}^{\dagger }{c}_{k}\\  &  & -{\rm{i}}\sum _{n=1}^{M}\,({J}_{n}^{x}-{J}_{n}^{y})\,\sin \,(nk)\,({c}_{-k}^{\dagger }{c}_{k}^{\dagger }+{c}_{-k}{c}_{k})+g\}\end{array}$$

by performing the Jordan-Wigner transformation in Eqs (3 and 4) and Fourier transformation in Eq. (5), respectively. We notice that the spinless fermion Hamiltonian is actually an extended one-dimensional mean field model for a triplet superconductor with long-range hopping.

An alternative way to diagonalize the Hamiltonian *H* is to induce the pseudo spin

19 $$\begin{array}{rcl}{s}_{k}^{-} & = & {({s}_{k}^{+})}^{\dagger }={c}_{k}{c}_{-k},\\ {s}_{k}^{x} & = & \frac{1}{2}\,({c}_{k}^{\dagger }{c}_{k}+{c}_{-k}^{\dagger }{c}_{-k}-1),\\ {s}_{k}^{z} & = & \frac{1}{2}\,({s}_{k}^{+}+{s}_{k}^{-}),\\ {s}_{k}^{y} & = & \frac{1}{2i}\,({s}_{k}^{+}-{s}_{k}^{-}),\end{array}$$

instead of Bogoliubov operator *γ*_*k*_. These operators satisfy the commutation relations of Lie algebra

20 $$[{s}_{k}^{x},{s}_{{k}^{^{\prime} }}^{\pm }]=\pm {\delta }_{k{k}^{^{\prime} }}{s}_{{k}^{^{\prime} }}^{\pm },[{s}_{k}^{+},{s}_{{k}^{^{\prime} }}^{-}]=2{\delta }_{k{k}^{^{\prime} }}{s}_{{k}^{^{\prime} }}^{x},$$

and lead to an alternative expression of the Hamiltonian

21 $$H=\sum _{k > 0}\,{H}_{k}=4\sum _{k > 0}\,\overrightarrow{B}\,(k)\cdot {\overrightarrow{s}}_{k},$$

where the components of $\overrightarrow{B}\,(k)$ are

22 $${B}_{x}=\sum _{n=1}^{M}\,({J}_{n}^{x}+{J}_{n}^{y})\,\cos \,(nk)-g,$$

23 $${B}_{y}=\sum _{n=1}^{M}\,({J}_{n}^{x}-{J}_{n}^{y})\,\sin \,(nk),$$

24 $${B}_{z}=0.$$

The spin of the operator ${\overrightarrow{s}}_{k}$ can be taken as *s*_*k*_ = 0, 0, and $\frac{1}{2}$ for four possible states, ${c}_{\pm k}^{\dagger }|0\rangle$, ${c}_{k}^{\dagger }{c}_{-k}^{\dagger }|0\rangle$ and |0〉. In this paper, we focus on the ground state, which corresponds to the case with ${s}_{k}=\frac{1}{2}$ for all *k*. In this sense, the physics of the Hamiltonian is clear, which represents an ensemble of spin$\mbox{-} \frac{1}{2}$ particles in the field of a magnetic monopole. We note that

25 $$[{H}_{k},{H}_{{k}^{^{\prime} }}]=\mathrm{0,}$$

which indicates that $\sum \,{H}_{k}$ for ${s}_{k}=\frac{1}{2}$ is equivalent to a Hamiltonian with two Bloch bands $\pm 2\,|\overrightarrow{B}\,(k)|$ ^25^.

In recent work^18^, it has been generally shown that a system as the form of Eq. (21) can be regarded as an ensemble of free spins on a loop subjected to a 2D magnetic field of Dirac monopole^26^. The variation of the groundstate energy density, which is a function of the loop, experiences a nonanalytical point when the winding number of the corresponding loop changes. This fact indicates the relation between quantum phase transition and the geometrical order parameter characterizing the phase diagram.

The concept of Berry phase can be introduced since *H*_*k*_ can be regarded as a parameter dependent Hamiltonian. Furthermore, when we consider the band under a slowly varying time-dependent perturbation, a quantized Berry phase should be obtained and may characterize the features of the band. As we shall see, the simplified Hamiltonian provides a natural platform to investigate the topological characterization of the QPT.

In the following, we consider two Hamiltonians *H*_ch_ + *H*_b_ and *H*_ch_, the ring Hamiltonian and the chain Hamiltonian. For the ring Hamiltonian, as mentioned above, the translational symmetry results in *H*_*k*_. This ensures the calculations of Chern and winding numbers, which are utilized to identify the quantum phase. For the chain Hamiltonian, we will transform *H*_ch_ into Majorana fermion representation. The phase diagram will be indicated by the number of zero modes.

### Chern and winding numbers

We note that the ring Hamiltonian *H*_*k*_ always connects a loop in an auxiliary space. In previous paper^18^, it has been shown that when the loop crosses the origin of the auxiliary space, phase transitions occur. Meanwhile, the winding number of the loop changes. Then the phase diagram can be characterized by the winding number of the loop. The conclusion is applicable to the present generalized model which corresponds to a loop tracing with the parametric equation $\overrightarrow{r}\,(k)=(x(k),y(k)\mathrm{,0})$ with

26 $$\{\begin{array}{l}x(k)=\sum _{n=1}^{M}\,({J}_{n}^{x}+{J}_{n}^{y})\,\cos \,(nk)-g\\ y(k)=\sum _{n=1}^{M}\,({J}_{n}^{x}-{J}_{n}^{y})\,\sin \,(nk)\end{array}.$$

The winding number of a closed curve in the auxiliary *xy*-plane around the origin is defined as

27 $${\mathscr{N}}=\frac{1}{2\pi }{\int }_{c}\,\frac{1}{{r}^{2}}\,(x{\rm{d}}y-y{\rm{d}}x),$$

which is an integer, representing the total number of times that the curve travels anticlockwise around the origin. Then we establish the connection between the QPT and the switch of the topological quantity. Here we present a class of simple models to illustrate the idea.

We consider a class of Hamiltonian indexed by *n*,

28 $$\begin{array}{rcl}{H}_{n} & = & \sum _{j=1}^{N}\,({J}_{n}^{x}{\sigma }_{j}^{x}{\sigma }_{j+n}^{x}+{J}_{n}^{y}{\sigma }_{j}^{y}{\sigma }_{j+n}^{y})\\  &  & \times \prod _{l=j+1}^{j+n-1}\,{\sigma }_{l}^{z}+g\sum _{j=1}^{N}\,{\sigma }_{j}^{z},\end{array}$$

which corresponds to a loop tracing with the parametric equation

29 $$\{\begin{array}{l}{x}_{n}(k)=({J}_{n}^{x}+{J}_{n}^{y})\,\cos \,(nk)-g\\ {y}_{n}(k)=({J}_{n}^{x}-{J}_{n}^{y})\,\sin \,(nk)\end{array}.$$

The geometry of the curve is obvious, which is the superposition of *n* identical ellipses with winding number ${\mathscr{N}}=n$ (−*n*) according to the Eq. (27) for $|g| < |{J}_{n}^{x}+{J}_{n}^{y}|$ and ${J}_{n}^{x2}-{J}_{n}^{y2} > 0$ $({J}_{n}^{x2}-{J}_{n}^{y2} < 0)$. Similarly, when we consider a Hamiltonian as *H*_*n*_ by switching ${J}_{n}^{x}$ and ${J}_{n}^{y}$ (or switching ${\sigma }_{j}^{x}$ and ${\sigma }_{j}^{y}$), the corresponding loop obeys the equation

30 $$\{\begin{array}{l}{x}_{n}(k)=({J}_{n}^{x}+{J}_{n}^{y})\,\cos \,(nk)-g\\ {y}_{n}(k)=-({J}_{n}^{x}-{J}_{n}^{y})\,\sin \,(nk)\end{array},$$

which still represents *n* identical ellipses but with winding number ${\mathscr{N}}=-n$ for $|g| < |{J}_{n}^{x}+{J}_{n}^{y}|$.

More explicitly, when we take ${J}_{n}^{y}={J}_{n\ne 1}^{x}=0$ and ${J}_{1}^{x}={J}^{x}\ne 0$, the system reduces to ordinary transverse field Ising model with Hamiltonians

31 $${H}_{{\rm{Ising}}}=\sum _{j=1}^{N}\,{J}^{x}{\sigma }_{j}^{x}{\sigma }_{j+n}^{x}+g\sum _{j=1}^{N}\,{\sigma }_{j}^{z}.$$

The winding number for the ground states of *H*_Ising_ with |*g*| < |*J* ^*x*^| is 1. Similarly, when taking ${J}_{n}^{x}={J}_{n\ne 1}^{y}=0$ and ${J}_{1}^{y}={J}^{y}\ne 0$, the system reduces to

32 $${H}_{{\rm{Ising}}}^{^{\prime} }=\sum _{j=1}^{N}\,{J}^{y}{\sigma }_{j}^{y}{\sigma }_{j+n}^{y}+g\sum _{j=1}^{N}\,{\sigma }_{j}^{z}.$$

The winding number for the ground states of ${H}_{{\rm{Ising}}}^{^{\prime} }$ with |*g*| < |*J* ^*y*^| is −1. The opposite signs represent two different quantum phases. These encouraging results strongly motivate further study of the relation between quantum phase and the geometric quantity of the system in the auxiliary space. To this end, we parameterize $\overrightarrow{r}\,(k)$ by its polar angle *θ* and azimuthal angle *φ*

33 $$\overrightarrow{r}\,(k,\phi )=(r\,\sin \,\phi \,\cos \,\theta ,r\,\sin \,\phi \,\sin \,\theta ,\,\cos \,\phi ),$$

where $$r=|\overrightarrow{r}\,(k)|=\sqrt{{x}^{2}\,(k)+{y}^{2}\,(k)}$$ and

34 $$\sin \,\theta =\frac{y\,(k)}{r},\,\cos \,\theta =\frac{x\,(k)}{r}.$$

It is a 2D-to-3D extension for the original model. The corresponding Hamiltonian can be expressed as

35 $${H}_{k}\,(\phi )=4\overrightarrow{r}\,(k,\phi )\cdot {\overrightarrow{s}}_{k},$$

which goes back to the original one when *φ* = *π*/2. The two eigenstates $|{u}_{k}^{\pm }\rangle$ with energies $\pm E\,=$ $\pm \sqrt{{\cos }^{2}\,\phi +{r}^{2}\,{\sin }^{2}\,\phi }$ are

36 $$|{u}_{k}^{\pm }\rangle =\frac{1}{\sqrt{2E\,(E\pm \,\cos \,\phi )}}\,(\begin{array}{c}\cos \,\phi \pm E\\ r{e}^{i\theta }\,\sin \,\phi \end{array}).$$

We are interested in the ground state, and then consider the lower energy level. The Berry connection is given by

37 $$\begin{array}{rcl}{A}_{k} & = & i\langle {u}_{k}^{-}|{\partial }_{k}|{u}_{k}^{-}\rangle \\  & = & -\frac{1}{2E\,(E-\,\cos \,\phi )}{r}^{2}\,{\sin }^{2}\,\phi \frac{\partial \theta }{\partial k},\end{array}$$

38 $$\begin{array}{rcl}{A}_{\phi } & = & i\,\langle {u}_{k}^{-}|{\partial }_{\phi }|{u}_{k}^{-}\rangle \end{array}=\mathrm{0,}$$

and the Berry curvature is

39 $${{\rm{\Omega }}}_{k\phi }={\partial }_{k}{A}_{\phi }-{\partial }_{\phi }{A}_{k}=-\frac{1}{2{E}^{3}}{r}^{2}\,\sin \,\phi \frac{\partial \theta }{\partial k}.$$

The corresponding Chern number is

40 $$\begin{array}{rcl}c & = & \frac{1}{2\pi }{\int }_{0}^{\pi }\,{\rm{d}}\phi \,{\int }_{0}^{2\pi }\,{\rm{d}}k{{\rm{\Omega }}}_{k\phi }\\  &  & -\frac{1}{4\pi }{\int }_{0}^{2\pi }\,{r}^{2}\frac{\partial \theta }{\partial k}{\rm{d}}k{\int }_{-1}^{1}\,{({r}^{2}-({r}^{2}-1){t}^{2})}^{-\mathrm{3/2}}\,{\rm{d}}t\\  &  & -\frac{1}{2\pi }\,[\theta (2\pi )-\theta (0)],\end{array}$$

where *t* = cos *φ*. It can be seen that the loop of a Hamiltonian is the intersection of the integral surface on the *xy* plane. Then we have the conclusion

41 $$|c|=|{\mathscr{N}}|.$$

Here we only take the equation for absolute values because the extension in Eq. (33) is not unique. There are many other ways of 2D-to-3D extension that can obtain the same result of Eq. (41). For example, one can take the extension by

42 $$\overrightarrow{r}\,(k,\phi )=|\overrightarrow{r}\,(k)|\,(\sin \,\phi \,\cos \,\theta ,\,\sin \,\phi \,\sin \,\theta ,\,\cos \,\phi ),$$

which leads to *c* = −*N*. However, after taking the transformation by replacement *θ* → −*θ* or *φ* → −*φ*, we have *c* = *N*. Then the relation between the signs of *c* and *N* depends on the way of the 2D-to-3D extension. Actually, the absolute sign of *c* or *N* is meaningless, while the relative sign of them is physical: opposite signs indicate two different states. For instance, the divergence of groundstate energy should occur when Chern number switches between ±1. The similar thing will happen in the Majorana charge of zero mode in next section.

### Majorana charge of zero mode

The above results indicate that the quantum phase of the model *H* exhibits topological characterization. Another way to unveil the hidden topology behind the model is exploring the zero modes of the corresponding Majorana Hamiltonian. Consider the system with open boundary conditions with the corresponding spinless fermion representation *H*_ch_ in Eq. (16).

We introduce Majorana fermion operators

43 $${a}_{j}={c}_{j}^{\dagger }+{c}_{j},{b}_{j}=-i\,({c}_{j}^{\dagger }-{c}_{j}),$$

which satisfy the relations

44 $$\{{a}_{j},{a}_{{j}^{^{\prime} }}\}=2{\delta }_{j,{j}^{^{\prime} }},\{{b}_{j},{b}_{{j}^{^{\prime} }}\}=2{\delta }_{j,{j}^{^{\prime} }},$$

45 $$\{{a}_{j},{b}_{{j}^{^{\prime} }}\}=0,{a}_{j}^{2}={b}_{j}^{2}=1.$$

The inverse transformation is

46 $${c}_{j}^{\dagger }=\frac{1}{2}\,({a}_{j}+i{b}_{j}),{c}_{j}=\frac{1}{2}\,({a}_{j}-i{b}_{j}).$$

Then the Majorana representation of the Hamiltonian is

47 $$H=i\sum _{n=1}^{M}\,\sum _{j=1}^{N-n}\,({J}_{n}^{x}{b}_{j}{a}_{j+n}-{J}_{n}^{y}{a}_{j}{b}_{j+n})+ig\sum _{j=1}^{N}\,{a}_{j}{b}_{j}.$$

We write down the Hamiltonian in the basis *ψ* ^*T*^ = (*a*_1_, *b*_1_, *a*_2_, *b*_2_, *a*_3_, *b*_3_, $\ldots$) and see that

48 $$H={\psi }^{T}h\psi ,$$

where *h* represents a 2*N* × 2*N* matrix. Here matrix *h* is explicitly written as

49 $$\begin{array}{rcl}h & = & \frac{i}{2}[\sum _{n=1}^{M}\,\sum _{l=1}^{N-n}\,({J}_{n}^{x}|2l\rangle \langle 2(l+n)-1|\\  &  & -{J}_{n}^{y}|2l-1\rangle \langle 2(l+n)|)+g\sum _{l=1}^{N}\,|2l-1\rangle \langle 2l|+{\rm{h}}.{\rm{c}}.],\end{array}$$

where basis $\{|l\rangle ,l\in [1,2N]\}$ is an orthonormal complete set, $\langle l|l^{\prime} \rangle ={\delta }_{l{l}^{^{\prime} }}$. By taking a local unitary transformation

50 $$i|2l\rangle \to |2l\rangle ,|2l-1\rangle \to |2l-1\rangle ,$$

we can express matrix *h* as a simpler form with real matrix elements,

51 $$\begin{array}{rcl}h & = & \frac{1}{2}[\sum _{n=1}^{M}\,\sum _{l=1}^{N-n}\,({J}_{n}^{x}|2l\rangle \langle 2\,(l+n)-1|\\  &  & +{J}_{n}^{y}|2l-1\rangle \langle 2\,(l+n)|)-g\sum _{l=1}^{N}\,|2l-1\rangle \langle 2l|+{\rm{h}}.{\rm{c}}.],\end{array}$$

which describes a tight-binding chain with long-range hopping.

We note that a winding number or Chern number with different signs denotes different phases. However, the number of zero modes is always positive, which is not precise to characterize the quantum phases difference. Here, for example, we consider two Hamiltonians

52 $${H}_{x}=-\sum _{j=1}^{N}\,{\sigma }_{j}^{x}{\sigma }_{j+1}^{x},{H}_{y}=-\sum _{j=1}^{N}\,{\sigma }_{j}^{y}{\sigma }_{j+1}^{y}.$$

The ground states can be obtained as

53 $$|{G}_{x}\rangle =\frac{1}{\sqrt{2}}(\prod _{j=1}^{N}\,{|\nearrow \rangle }_{j}+\prod _{j=1}^{N}\,{|\swarrow \rangle }_{j}),$$

54 $$|{G}_{y}\rangle =\frac{1}{\sqrt{2}}(\prod _{j=1}^{N}\,{|\nwarrow \rangle }_{j}+\prod _{j=1}^{N}\,{|\searrow \rangle }_{j}),$$

where ${\sigma }_{j}^{x}{|\nearrow \rangle }_{j}={|\nearrow \rangle }_{j}$, ${\sigma }_{j}^{x}{|\swarrow \rangle }_{j}=-{|\swarrow \rangle }_{j}$, ${\sigma }_{j}^{y}{|\nwarrow \rangle }_{j}={|\nwarrow \rangle }_{j}$, and ${\sigma }_{j}^{y}{|\searrow \rangle }_{j}=-{|\searrow \rangle }_{j}$. Two states obviously belong to two different quantum phases in the context of the original Hamiltonian. However, the number of edge states of the corresponding fermion models are both 2.

It is expected to have another quantity which allows us to discern two phases with opposite Chern numbers to replace the number of zero modes. To this end, we introduce the concept of Majorana charge for the first time to characterize the quantum phase. The magnitude of a Majorana charge is defined by the distribution of particle probability in zero-mode states, and the sign is defined by the type of Majorana fermions, *a*_*j*_ or *b*_*j*_. The exact expression of a Majorana charge is

55 $$ {\mathcal M} =\sum _{\alpha }\,\langle \alpha |{\widehat{M}}_{+}-{\widehat{M}}_{-}|\alpha \rangle ,$$

where $|\alpha \rangle$ denotes the zero mode state, and ${\widehat{M}}_{\pm }$ denotes the particle number operator of Majorana fermion, which is defined as

56 $$\begin{array}{r}{\widehat{M}}_{+}=\sum _{l=1}^{{N}_{+}}(|2l-1\rangle \langle 2l-1|\,+\,|2N+2-2l\rangle \langle 2N+2-2l|)\end{array}$$

57 $$\begin{array}{r}{\widehat{M}}_{-}=\sum _{l=1}^{{N}_{-}}\,(|2l\rangle \langle 2l|\,+\,|2N+1-2l\rangle \langle 2N+1-2l|),\end{array}$$

with

58 $${N}_{\pm }=\frac{N}{2}\pm \frac{1+{(-1)}^{N+1}}{4}.$$

For even or large *N* case, one can simply take *N*_±_ = *N*/2.

Now we focus on the relation between ${\mathcal M}$ and Chern number. We start our investigation from simple cases with ${J}_{n}^{y}={J}_{n\ne {n}_{0}}^{x}=0$ and *g* = 0. The corresponding loop in the auxiliary space reduces to

59 $$\{\begin{array}{l}{x}_{{n}_{0}}\,(k)={J}_{{n}_{0}}^{x}\,\cos \,({n}_{0}k)\\ {y}_{{n}_{0}}\,(k)={J}_{{n}_{0}}^{x}\,\sin \,({n}_{0}k)\end{array},$$

which is the superposition of *n*_0_ identical circles with winding number ${\mathscr{N}}={n}_{0}$. The corresponding matrix in Majorana fermion representation is

60 $${h}_{{n}_{0}}=\frac{{J}_{{n}_{0}}^{x}}{2}(\sum _{l=1}^{N-{n}_{0}}\,|2l\rangle \langle 2\,(l+{n}_{0})-1|+{\rm{h}}.{\rm{c}}.).$$

**Figure 2:**
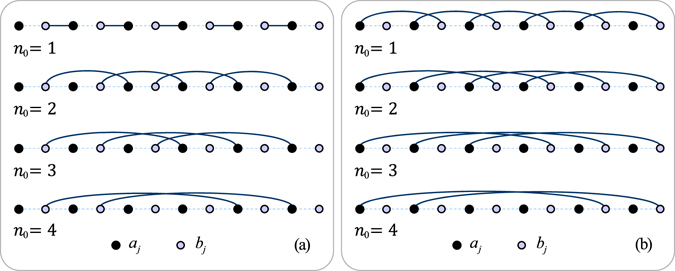
The structures of Majorana lattices for ${h}_{{n}_{0}}$ defined in Eq. (60) with *n*_0_ = 1, 2, 3, 4. We find that ${h}_{{n}_{0}}$ contains 2*n*_0_ isolated sites, allowing the existence of zero modes. The isolated sites appear every other sites. But in fig (**a**), the isolated sites appear starting from the outermost sites; in fig (**b**), the isolated sites appear starting from the secondary outermost sites.

Obviously, *h*_1_ describes a dimerized chain, which possesses two zero modes and Majorana charge ${\mathcal M} =2$. Moreover, it can be shown that the spectrum of ${h}_{{n}_{0}}$ possesses 2*n*_0_ zero modes and the Majorana charge ${\mathcal M} =2{n}_{0}$. Based on the above analysis, we know that when we consider other simple cases with ${J}_{n}^{x}={J}_{n\ne {n}_{0}}^{y}=0$ and *g* = 0. The corresponding loop in the auxiliary space reduces to

61 $$\{\begin{array}{l}{x}_{{n}_{0}}\,(k)={J}_{{n}_{0}}^{y}\,\cos \,({n}_{0}k)\\ {y}_{{n}_{0}}\,(k)=-{J}_{{n}_{0}}^{y}\,\sin \,({n}_{0}k)\end{array},$$

which has winding number −*n*_0_. On the other hand, we have

62 $${\overline{h}}_{{n}_{0}}=-\frac{{J}_{{n}_{0}}^{y}}{2}(\sum _{l=1}^{N-{n}_{0}}\,|2l-1\rangle \langle 2\,(l+{n}_{0})|+{\rm{h}}.{\rm{c}}.),$$

which can show that the spectrum of ${\overline{h}}_{{n}_{0}}$ possesses 2*n*_0_ zero modes and the Majorana charge ${\mathcal M} =-2{n}_{0}$. The structure of Majorana lattices for ${h}_{{n}_{0}}$ and ${\overline{h}}_{{n}_{0}}$ with *n*_0_ = 1, 2, 3, and 4 are schematically illustrated in Fig. 2.

These situations correspond to fined-tuned points, in which the eigenstates of zero-mode can be obtained exactly. Different values of ${\mathcal M}$ should identify different quantum phases. It is true for the cases beyond the fined-tuned points. To demonstrate this idea, we compute the phase diagram of a toy model with parameters satisfying the equations

63 $${J}_{n}^{x}=\exp \,\{-4{[x-\frac{1}{2}(3-n)]}^{2}\},$$

64 $${J}_{n}^{y}=2\,(y-1)\,\exp \,\{-4{[y-\frac{1}{2}(2-n)]}^{2}\}.$$

**Figure 3:**
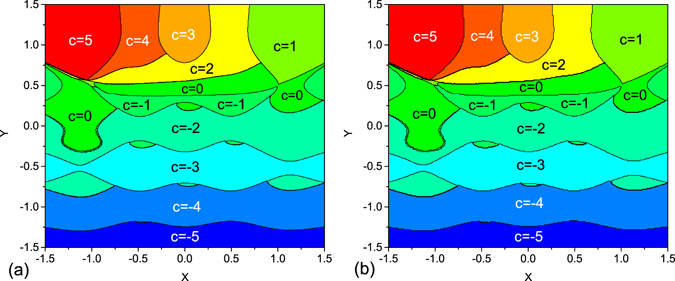
Phase diagrams for system with parameters satisfying the equation (40) identified by Chern numbers. The Chern number is obtained by two ways: (**a**) It is computed by the winding numbers from formula in Eq. (27). (**b**) It is computed by the number of zero modes with the sign of Majorana charge defined in Eq. (55). The result is obtained by exact diagonalization for Hamiltonian in Eq. (51) on *N* = 200 chain. The units of x and y are the same as that of *J*_*n*_, which is taken as dimensionless unit.

The phase diagrams are obtained by Chern numbers for given $({J}_{n}^{x},{J}_{n}^{y})$, which are computed in two different ways. On the one hand, one can calculate the winding number through the numerically integration in Eq. (27), which has been shown to be equal to Chern number. On the other hand, one can figure out the number of zero modes by exact diagonalization of the Majorana matrix for finite *N*. The sign of Chern number can be determined by the sign of the corresponding Majorana charge defined in Eq. (55). In Fig. 3 the phase diagrams obtained by two methods are plotted, which is in accord with our predictions.

We have established our main results, and a few comments are in order. First, notice that the zero mode states calculated here are not at exact zero-energy for finite system except the cases for Hamiltonians in Eqs (60) and (62). Accordingly, the Majorana charges are not located at exact edges. Second, we would like to point out that the sign of ${\mathcal M}$ is not absolute, but depend on the definition of ${\mathcal M}$. If we take the definition of ${\mathcal M}$ by switching the positions of ${\widehat{M}}_{+}$ and ${\widehat{M}}_{-}$, i.e., ${\widehat{M}}_{\pm }\to {\widehat{M}}_{\mp }$, we will have the result with opposite sign of ${\mathcal M}$ i.e., ${\mathcal M} \to - {\mathcal M}$. Thus we say that the sign of ${\mathcal M}$ for a given quantum phase is not definite. However, the relative sign of Majorana charge is meaningful: different signs indicate different phases. Then when we say $c=N= {\mathcal M} \mathrm{/2}$, a suitable 2D-to-3D extension and Majorana representation should be chosen.

### Invariance under perturbations

The above analysis demonstrated that the quantum phase transitions in the spin system can be characterized by the numbers of ground state, such as *c*, ${\mathscr{N}}$ and ${\mathcal M}$, in stead of order parameter of spontaneous symmetry breaking. In general, these numbers capture the topological structure of the ground state wave function. In principle, topology is a global property that cannot be changed by a small perturbations to the system. However, the robustness of the topological quantity depends on the type of perturbation in concrete systems. In this section, we exemplify this point by two examples.

We start by the Hamiltonian

65 $$h={h}_{0}+h^{\prime} $$

66 $$\begin{array}{r}{h}_{0}=\frac{1}{2}{J}_{1}^{x}(\sum _{l=1}^{N-1}\,|2l\rangle \langle 2l+1|-\lambda \sum _{l=1}^{N}\,|2l-1\rangle \langle 2l|\,+\,{\lambda }^{N}|2N\rangle \langle 1|+{\rm{h}}.{\rm{c}}.),\end{array}$$

67 $$h^{\prime} =\sum _{n=1}^{{n}_{0}}\,\sum _{l=1}^{N-n}\,{{\rm{\Delta }}}_{n}(|2l-1\rangle \langle 2l+2n|).$$

Here *h*_0_ is the Majorana representation of a Kitaev chain, which is derived from Ising chain with $\lambda =g/{J}_{1}^{x}$. There is an additional term acrossing two ends of the chain, which will vanish in thermodynamic limit for *λ* < 1 and *h*_0_ goes back to a standard SSH chain. *h*′ presents long-range interaction. It is well known that there are two zero modes for *λ* < 1 in thermodynamic limit, which is a topological feature of the quantum phase. It is interesting to investigate what happens of the zero mode of *h*_0_ under the perturbation *h*′.

We will show the robustness of the topology of the Kitaev chain based on exact solution. A straightforward derivation^27^ shows that two states

68 $$\{\begin{array}{l}|{{\rm{\Psi }}}_{1}\rangle =\frac{1}{\sqrt{{\rm{\Omega }}}}\sum _{l=1}^{N}\,{\lambda }^{l-1}|2l-1\rangle ,\\ |{{\rm{\Psi }}}_{2}\rangle =\frac{1}{\sqrt{{\rm{\Omega }}}}\sum _{l=1}^{N}\,{\lambda }^{N-l}|2l\rangle ,\end{array}$$

where Ω = (1 − *λ* ^2*N*^)/(1 − *λ* ^2^) is normalization factor, are eigenstates of *h*_0_ for finite *N* with zero energy. For finite *N*, *h*′ splits the degeneracy from zero energy to *E* ^(1)^, which can be estimated by degenerate perturbation method. The matrix of *h*′ on $\{|{{\rm{\Psi }}}_{1}\rangle ,|{{\rm{\Psi }}}_{2}\rangle \}$ is

69 $$h^{\prime} =\frac{1}{{\rm{\Omega }}}\sum _{n=1}^{{n}_{0}}\,{{\rm{\Delta }}}_{n}\,(N-n)\,{\lambda }^{N-1-n}\,(\begin{array}{cc}0 & 1\\ 1 & 0\end{array}),$$

which yields

70 $$\begin{array}{rcl}{E}^{\mathrm{(1)}} & = & \pm \frac{1}{{\rm{\Omega }}}\sum _{n=1}^{{n}_{0}}\,{{\rm{\Delta }}}_{n}\,(N-n)\,{\lambda }^{N-1-n}\\  & = & \pm \frac{{\lambda }^{N-1}}{{\rm{\Omega }}}\,\sum _{n=1}^{{n}_{0}}\,{{\rm{\Delta }}}_{n}\,(N-n)\,{\lambda }^{-n}.\end{array}$$

Obviously, *E* ^(1)^ tends to zero in thermodynamic limit, i.e., the zero modes are invariant under the perturbations.

Next we consider other two cases with Hamiltonians

71 $${h}_{a}={h}_{2}-\frac{\lambda }{2}\sum _{l=1}^{N}\,|2l-1\rangle \langle 2l|+{\rm{h}}.{\rm{c}}.$$

72 $$\begin{array}{rcl}{h}_{b} & = & {h}_{2}+\frac{\lambda }{2}(\sum _{l=1}^{N-1}\,|2l\rangle \langle 2(l+1)-1|\\  &  & -\sum _{l=1}^{N}\,|2l-1\rangle \langle 2l|)+{\rm{h}}.{\rm{c}}.,\end{array}$$

where ${h}_{2}={h}_{{n}_{0}}\,({n}_{0}=2)$ in Eq. (60). It has been shown that *h*_2_ has four zero modes for finite *N*. It is interesting to investigate what happens of the zero modes under the *λ*-term perturbations.

**Figure 4:**
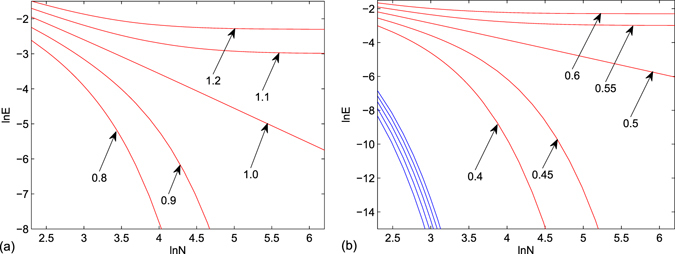
Positive energy levels related to zero modes for *N*-site systems (**a**) *h*_*a*_ and (**b**) *h*_*b*_ with typical values of *λ*, calculated by exact diagonalization. (**a**) Two positive levels of *h*_*a*_ are degenerate, which turns to vanish for *λ* < 1, is a straight line for *λ* = 1, and converge to a finite value for *λ* > 1. (**b**) One of two positive levels (blue) turns to vanish for given *λ*, while another level has the similar behavior as that in (**a**) when *λ* are around 0.5.

In this situation, analytical analysis cannot provide definite information. We compute the eigen levels near the zero point for systems with different *λ* and *N* by exact diagonalization. In both two systems, the eigen levels are symmetric about the zero. Thus we only consider two positive levels. The profile of energy as functions of *λ* and *N* is plotted in Fig. 4. We find that, (i) two positive levels of *h*_*a*_ are degenerate. Their behavior at large *N* depends on *λ*: when *λ* < 1, the energy turns to zero, while converges to a finite value for *λ* > 1; (ii) two positive levels of *h*_*b*_ are not degenerate. One of the two always turns to zero for both *λ* > 0.5 and *λ* < 0.5. Another level at large *N* depends on *λ*: when *λ* < 0.5, the energy turns to zero, while converges to a finite value for *λ* > 0.5. It indicates that in large-*N* limit, four zero modes are robust unless occur QPT.

## Discussion

We have studied the topological characterization of QPTs in a family of exactly solvable Ising models with short- and long-range interactions. We have calculated the Chern number and winding number for the models with periodic boundary conditions. We have shown exactly that the Chern number and winding number are identical and can be utilized to characterize the phase diagram. This conclusion is applicable for more generalized systems. We have also calculated the Majorana mode charge analytically and numerically. Our results indicate that the three numbers are equivalent. Although our conclusion is obtained for specific models, it reveals the possible connection between traditional and topological QPTs.

## Method

### Winding and Chern numbers

We would like to point out that this conclusion (41) is true for any models in the form ${H}_{k}\propto x{s}_{x}^{k}+y{s}_{y}^{k}$. For the model in Eq. (1), the corresponding parameter equations of the integral surfaces are

73 $$\{\begin{array}{l}x=r\,\sin \,\phi \,\cos \,\theta ,\\ y=r\,\sin \,\phi \,\sin \,\theta ,\\ z=\,\cos \,\phi ,\end{array}$$

where polar angle *θ* and radius *r* are explicitly expressed as

74 $$\tan \,\theta =\frac{{\sum }_{n=1}^{M}({J}_{n}^{x}-{J}_{n}^{y})\,\sin \,(nk)}{{\sum }_{n=1}^{M}({J}_{n}^{x}+{J}_{n}^{y})\,\cos \,(nk)-g},$$

75 $$\begin{array}{rcl}r & = & \{{[\sum _{n=1}^{M}({J}_{n}^{x}-{J}_{n}^{y})\sin (nk)]}^{2}\\  &  & {+{[\sum _{n=1}^{M}({J}_{n}^{x}+{J}_{n}^{y})\cos (nk)-g]}^{2}\}}^{\mathrm{1/2}}.\end{array}$$

**Figure 1:**
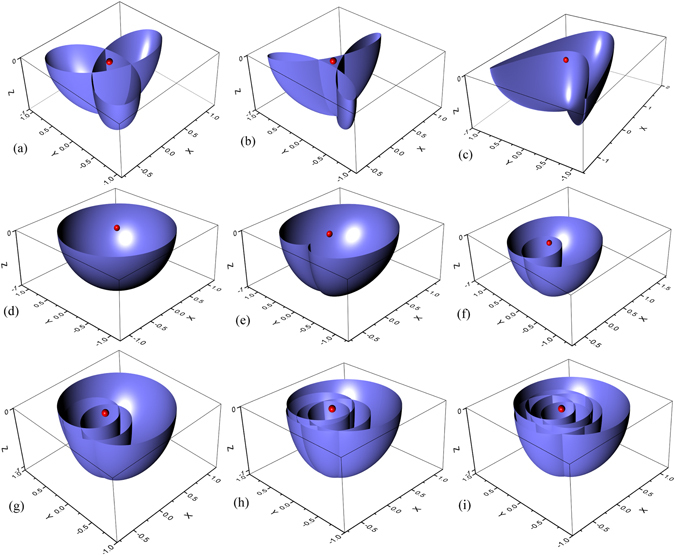
Plots of the surfaces in the auxiliary space (*x*, *y*, *z*) to illustrate the relation between winding and Chern number. The corresponding parameters in equations of the 3D surfaces are listed in Table 1. The red dot denotes the origin of the auxiliary space (0, 0, 0). The winding numbers can be figured out from the curves in the *xy* plane.

In this paper, we illustrate our conclusion by several typical cases with the values of ${J}_{n}^{x}$ and ${J}_{n}^{y}$ (*n* ∈ $[1,5]$) listed in Table 1 and plot the 3D surfaces in Fig. 1. The plots display the relation between the magnitudes of winding and Chern number clearly. However, the signs of the numbers cannot be visualized in the plots. This can be done by tracing the plots for varying *k*.

**Table 1:** Typical examples illustrating the relations among winding numbers, zero modes and Majorana charges.

FI	*N*	${{\boldsymbol{J}}}_{{\bf{1}}}^{{\boldsymbol{x}}}$, ${{\boldsymbol{J}}}_{{\bf{1}}}^{{\boldsymbol{y}}}$	${{\boldsymbol{J}}}_{{\bf{2}}}^{{\boldsymbol{x}}}$, ${{\boldsymbol{J}}}_{{\bf{2}}}^{{\boldsymbol{y}}}$	${{\boldsymbol{J}}}_{{\bf{3}}}^{{\boldsymbol{x}}}$, ${{\boldsymbol{J}}}_{{\bf{3}}}^{{\boldsymbol{y}}}$	${{\boldsymbol{J}}}_{{\bf{4}}}^{{\boldsymbol{x}}}$, ${{\boldsymbol{J}}}_{{\bf{4}}}^{{\boldsymbol{y}}}$	${{\boldsymbol{J}}}_{{\bf{5}}}^{{\boldsymbol{x}}}$, ${{\boldsymbol{J}}}_{{\bf{5}}}^{{\boldsymbol{y}}}$	*N*_*zm*_	*M* (*N* = 50, 200)
a	−2	0.4, 0	0, 0.6	0, 0	0, 0	0, 0	4	−3.9912, −4.0000
b	−1	0, 0.55	0.45, 0	0, 0	0, 0	0, 0	2	−1.8963, −2.0000
c	0	0.8, −0.2	0.5, 0.5	0, 0	0, 0	0, 0	0	0, 0
d	1	1, 0	0, 0	0, 0	0, 0	0, 0	2	2.0000, 2.0000
e	1	0.8, 0	0.4, 0	0, 0	0, 0	0, 0	2	2.0000, 2.0000
f	2	0.4, 0	0.6, 0	0, 0	0, 0	0, 0	4	4.0000, 4.0000
g	3	0.3, 0	0.2, 0	0.5, 0	0, 0	0, 0	6	6.0000, 6.0000
h	4	0.25, 0	0.1, 0	0.15, 0	0.5, 0	0, 0	8	7.9997, 8.0000
i	5	0.2, 0	0, 0	0.15, 0	0.15, 0	0.5, 0	10	9.9938, 10.0000

The values of ${J}_{n}^{x}$ and ${J}_{n}^{y}$ (*n* ∈ $[1,5]$) are the parameters for equations of plots in Fig. 1 with figure index (FI) (a)–(i) and corresponding numbers ${\mathscr{N}}$. For finite size systems with *N* = 50 and 200, the zero modes and Majorana charges are obtained by exact diagonalizations. We define the zero modes by selecting eigenstates with absolute eigenvalues less than 10^−3^. *N*_zm_ is the number of such eigenstates for every cases. The Majorana charges are calculated from Eq. (55) for given zero mode states. We can see that ${N}_{{\rm{zm}}}=2|{\mathscr{N}}|$ and ${\mathcal M}$ closes to $2{\mathscr{N}}$ as *N* increases.

### Zero mode and Majorana charge

For the cases with ${J}_{n}^{y}={J}_{n\ne {n}_{0}}^{x}=0$ and *g* = 0, the matrix in Majorana fermion representation is

76 $${h}_{{n}_{0}}=\frac{{J}_{{n}_{0}}^{x}}{2}(\sum _{l=1}^{N-{n}_{0}}\,|2l\rangle \langle 2\,(l+{n}_{0})-1|+{\rm{h}}.{\rm{c}}.).$$

Straightforward derivations show that

77 $$[|2j-1\rangle \langle 2j-1|,{h}_{{n}_{0}}]=\mathrm{0,}$$

for *j* < *n*_0_ + 1, and

78 $$[|2j\rangle \langle 2j|,{h}_{{n}_{0}}]=0,$$

for *j* > *N* − *n*_0_. This indicates that there are always 2*n*_0_ isolated sites in the ending region of the chain, resulting in 2*n*_0_ eigenstates with zero energy. This analysis is applicable for ${\overline{h}}_{{n}_{0}}$. In both situations, the Majorana charge is equal to the winding number and Chern number. In Fig. 2. the structures of Majorana lattices for ${h}_{{n}_{0}}$ and ${\overline{h}}_{{n}_{0}}$ with *n*_0_ = 1, 2, 3, and 4 are schematically illustrated. We find that ${h}_{{n}_{0}}$ and ${\overline{h}}_{{n}_{0}}$ contain 2*n*_0_ isolated sites, allowing the existence of zero modes. In the present stage, we cannot provide a proof for general case. We explore the general case by exact numerical simulations. We calculate the eigenvalues and Majorana charges for the systems with the parameters listed in Table 1 (a)–(i) for finite *N*. Numerical results indicate that the Majorana charges accord with the winding numbers or Chern numbers.
